# A Systematic Review of Methods and Procedures Used in Ecological Momentary Assessments of Diet and Physical Activity Research in Youth: An Adapted STROBE Checklist for Reporting EMA Studies (CREMAS)

**DOI:** 10.2196/jmir.4954

**Published:** 2016-06-21

**Authors:** Yue Liao, Kara Skelton, Genevieve Dunton, Meg Bruening

**Affiliations:** ^1^ University of Texas MD Anderson Cancer Center Department of Behavioral Science Houston, TX United States; ^2^ University of Alabama, Birmingham Birmingham, AL United States; ^3^ University of Southern California Department of Preventive Medicine Los Angeles, CA United States; ^4^ Arizona State University School of Nutrition and Health Promotion Phoenix, AZ United States

**Keywords:** ecological momentary assessment, nutrition, physical activity, youth, systematic review, reporting checklist

## Abstract

**Background:**

Ecological momentary assessment (EMA) is a method of collecting real-time data based on careful timing, repeated measures, and observations that take place in a participant’s typical environment. Due to methodological advantages and rapid advancement in mobile technologies in recent years, more studies have adopted EMA in addressing topics of nutrition and physical activity in youth.

**Objective:**

The aim of this systematic review is to describe EMA methodology that has been used in studies addressing nutrition and physical activity in youth and provide a comprehensive checklist for reporting EMA studies.

**Methods:**

Thirteen studies were reviewed and analyzed for the following 5 areas of EMA methodology: (1) sampling and measures, (2) schedule, (3) technology and administration, (4) prompting strategy, and (5) response and compliance.

**Results:**

Results of this review showed a wide variability in the design and reporting of EMA studies in nutrition and physical activity among youth. The majority of studies (69%) monitored their participants during one period of time, although the monitoring period ranged from 4 to 14 days, and EMA surveys ranged from 2 to 68 times per day. More than half (54%) of the studies employed some type of electronic technology. Most (85%) of the studies used interval-contingent prompting strategy. For studies that utilized electronic devices with interval-contingent prompting strategy, none reported the actual number of EMA prompts received by participants out of the intended number of prompts. About half (46%) of the studies failed to report information about EMA compliance rates. For those who reported, compliance rates ranged from 44-96%, with an average of 71%.

**Conclusions:**

Findings from this review suggest that in order to identify best practices for EMA methodology in nutrition and physical activity research among youth, more standardized EMA reporting is needed. Missing the key information about EMA design features and participant compliance might lead to misinterpretation of results. Future nutrition and physical activity EMA studies need to be more rigorous and thorough in descriptions of methodology and results. A reporting checklist was developed with the goal of enhancing reliability, efficacy, and overall interpretation of the findings for future studies that use EMAs.

## Introduction

The number of overweight or obese youth in the United States is alarming for public health professionals, as prevalence of overweight/obesity among youth is estimated to be 31.8% [1]. National data suggests that only 15.7% of adolescents ate vegetables 3 or more times during the past 7 days and only 29% of adolescents achieved 60 minutes of physical activity per day [2]. US children and adolescents’ lifestyle factors, such as poor diet and physical inactivity, are related to an increased risk for chronic diseases, including diabetes, hypertension, cardiovascular diseases, and other metabolic disorders [3,4]. Many current methods for assessing nutrition and physical activity (eg, dietary recalls, physical activity logs) are limited since they can introduce high participant burden [5] and are prone to inaccuracies. More studies that use assessment methods that may limit participant burden and provide ecologically valid data for nutrition and physical activity behaviors are needed.

Advances in electronic technologies and societal changes have created opportunities to assess youth nutrition and physical activity behaviors as they occur in their daily lives. Real-time data capture methods refer to collecting data as it naturally occurs [6]. Real-time data assessments differ from traditional retrospective data collection methods as they sample snapshots of participants’ lives to capture the variability of experiences more accurately. As information is collected at or near the moment when events and experiences occur, real-time data capture methods can reduce memory and other biases that are associated with retrospective recall measures [7]. Ecological momentary assessment (EMA), a type of real-time data capture method, was originally developed for psychological assessments of mood and affect [8]. Shiffman and colleagues [5] define EMA as “monitoring or sampling strategies to assess phenomena at that moment they occur in natural settings.” There are several unique features common to the EMA methods: (1) the data capture happens in subjects’ natural environment—the “Ecological” aspect of EMAs; (2) assessments focus on current feelings and behaviors, rather than concentrating on recall or summary over long periods of time—the “Momentary” aspect of EMAs; (3) the moments are assessed by random sampling, event-based sampling, interval sampling, or a combination of any of these strategies; and (4) multiple assessments are collected over time to provide a profile for behavior throughout time—the “Assessment” aspect of EMAs [5].

Nutrition and physical activity studies that employ the EMA methodology enable the collection of data with an array of variables including behavioral, physical, sociopsychological, and contextual information [8]. This assessment strategy makes it possible to examine concurrent exposures and events, such as examining where and with whom physical activity and sedentary behavior are likely to occur during the course of participants’ everyday lives [9]. Due to the repeated measurements used in EMA methodology, EMA studies are able to focus on within-person changes in behaviors and experiences over time, thus allowing the investigation of antecedents and consequences of a behavior [10], and the advanced modeling of how variation in momentary cognitive state might relate to behaviors [11].

Over the past several years, there has been an increase in the popularity and prevalence of research conducted using EMAs. Given the potential methodological and analytical advantages of using EMAs in nutrition and physical activity research in youth, this review is aimed to describe features of EMA methodology in studies that address nutrition and physical activity in children and adolescents. In addition, although some guidance is available for designing and reporting in EMA studies [12], there are currently no specific guidelines for the necessary detail in reporting in EMA studies, which could make a systematic synthesis of results from EMA studies challenging. Similar reporting checklists for other types of studies have been widely adopted. For example, the Strengthening the Reporting of Observational Studies in Epidemiology (STROBE) is a commonly used checklist of items for observational studies [13]. It contains 22 items that relate to the title, abstract, introduction, methods, results, and discussion sections of papers with the goal to improve the quality of reporting. Building on the STROBE checklist and the EMA design guidelines by Stone and Shiffman [12], a comprehensive checklist of specific items to be reported for EMA studies was also developed: Checklist for Reporting EMA Studies (CREMAS).

## Methods

### Information Sources

CINAHL, PsychINFO, PubMed, and EBSCOhost were searched for relevant studies that were published before July 2015. The keywords used included “ecological momentary assessment” and “EMA,” in combination with “food,” “nutrition,” “eating,” “food consumption,” “eating habits,” “physical activity; PA,” “text messaging,” “SMS telephone,” “electronic diaries,” and “prompting.” A hand search of the reference section of all papers was conducted to review for additional papers that were missed during the electronic search.

### Selection Criteria

Inclusion criteria for this review were as follows: (1) published in English, (2) used EMA-based data collection method, (3) had a mean participant age of 22 years or younger (or enrolled in a college/university), and (4) focused on the assessment of nutrition or physical activity habits. Studies were excluded if they did not have repeated measures, did not assess variable/outcome measures via EMAs, had a mean participant age greater than 22 years, assessed maladaptive or disordered nutrition or physical activity behaviors, and/or were intervention studies. Further, papers must have reported results of EMAs; papers that only described EMA design were not included.

### Data Extraction

Data were extracted in two passes. In the first pass, data pertaining to the following general study characteristics were extracted from each of the studies: sample size, study design, measures, research questions/objectives, findings, and limitations/future directions. In the second pass, data extraction continued by gathering specific methodological features and response- and compliance-related information. In particular, data were synthesized from the following 5 main areas:

1. *Sampling and measures*: sample characteristics and tools used in the EMA protocol

2. *Schedule*: monitoring periods (number of waves from which data were collected), duration (number of days that each monitoring period lasted), prompt frequency (frequency of EMA prompts per day), and prompt interval (the time between each EMA prompt)

3. *Technology and administration*: use or lack of technology and method of administration of EMAs

4. *Prompting strategy*: methods used to cue participants—interval contingent (EMA prompts were set for certain intervals that were not random), random interval contingent (EMA prompts were set to be randomized throughout the day), event based (EMAs were recorded when eating occasions or physical activity occurred), or evening report (EMAs administered in the evenings to summarize the events of the day)

5. *Response and compliance*: participation rate, gathered data, missing data (ie, unanswered and/or unprompted EMA surveys), latency (ie, the time period between when participants receive an EMA prompt and when the EMA is answered), and attrition (ie, the number of participants who dropped out of the study for any reason).

For studies that did not report any of this data, calculations were performed using information provided in the paper whenever possible.

A coding form was developed based on the above areas of interest and two raters extracted information from each study independently for all items. Agreement among raters for each item ranged from 85 to 100%, and all discrepancies were resolved through discussions that led to consensus.

## Results

### Literature Search

**Figure 1:**
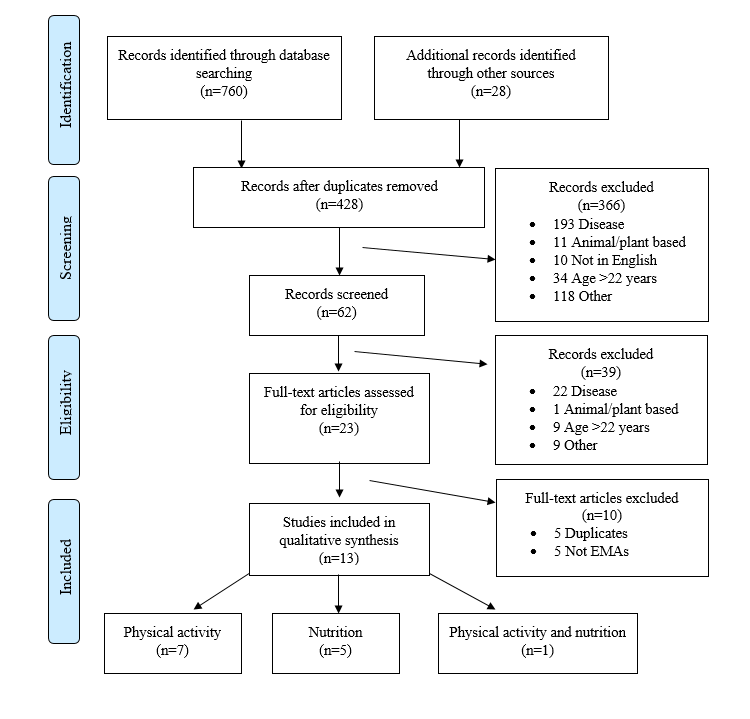
PRISMA Flow Diagram for paper selection process.

After completing a systematic review of databases and reference lists, a total of 428 potentially relevant studies were screened. From this group, 62 article abstracts were identified and evaluated for inclusion criteria, and 23 were selected for further full-text review. In cases where multiple papers were published (*n*=5) on the same study (eg, reporting validity, reporting outcomes, translations, etc.), information was extracted from all papers and presented as a single study. On the basis of the abovementioned criteria, 13 independent papers were retained for inclusion in the review, 7 studies were physical activity-related, 5 focused on nutrition outcomes, and 1 study assessed both physical activity and nutrition behaviors. Figure 1 presents a flow chart of the systematic literature search, according to the Preferred Reporting Items for Systematic Reviews and Meta-Analyses (PRISMA) guidelines.

### Sampling and Measures

Sample characteristics and methodological features of each included study are presented in Table 1. The mean number of participants per study was 391 (range=30-1604, median=147). However, this mean is skewed by 5 studies with samples over 500 [14-18]. Excluding these 5 studies, the mean sample size was 82 (range=30-158, median=63). Two studies did not report mean age [19,20]. Excluding those 2 studies, the mean sample-weighted age of participants was 15.6 years, with a range of 5.3-21.0 years.

One study asked participants to respond to the question, “What are you doing now?” All other studies used retrospective questions (ranging from every 15 minutes to 4 hours) to assess nutrition and physical activity behaviors. Only one study combined EMA with an objective measurement (ie, accelerometry) [21].

**Table 1:** Methodological features of ecological momentary assessment (EMA) nutrition and physical activity studies in youth.

Citation	Technology^a^	Prompt approach^b^	Monitoring periods^c^	Duration (days) per monitoring period^d^	Prompt frequency per day^e^	Prompt interval^f^
Berkman et al [22]	Paper-and-pencil diary and cell phone	Event-based	1	14	4	Breakfast, lunch, dinner, and bedtime predefined by participants
Biddle et al [14]	Paper-and-pencil diary	Fixed interval contingent	1	4	44 weekdays 68 weekends	15 minutes
Biddle et al [15]	Paper-and-pencil diary	Interval contingent	1	4	44 weekdays 68 weekends	15 minutes
Carels et al [23]	Paper-and-pencil diary	Event-based & random interval contingent	1	7	-	<15 minutes of event
Dunton et al [16]	Palm III handheld computer	Fixed interval contingent	8	4	20-30	30 minutes (+ 10 minutes)
Dunton et al [24]	HTC Shadow cell phone	Random interval contingent	2	4	3 weekdays 7 weekends	Random within 2-hour blocks
Gorely et al [17]	Paper-and-pencil diary	Fixed interval contingent	2	4	44 weekdays 68 weekends	15 minutes
Grenard et al [25]	Palm E2 PCA handheld computer	Event-based, fixed interval contingent, and evening report	1	7	2 weekday 4 weekends	Event-based: < 15 minutes of event Fixed interval: 3 hours
Mak et al [18]	Paper-and-pencil diary	Event-based	1	4	7	3 hours
Rouse et al [26]	Paper-and-pencil diary	Fixed interval contingent	1	2	44 weekdays 68 weekends	15 minutes
Rusby et al [19]	iPod touch handheld computer	Random interval contingent	4	7	3 M-T 4 F 6 Sat. 5 Sun	90-120 minutes
Spook et al [20]	Blackberry OS, Android, iOS, mobile phones	Event-based and interval contingent	1	7	5	3-4 hours
Thomas et al [27]	Palm-top handheld computer	Random interval contingent	1	7	6	Variable

^a^Technology: operating system, device type, and/or phone model (in as much detail as was provided in the paper).

^b^Prompt approach: type of EMA sampling.

^c^Monitoring periods: number of waves EMA was used in the study.

^d^Duration: number of days each monitoring period lasted.

^e^Prompt frequency: number of times it was intended for participants to answer EMA prompts.

^f^Prompt interval: time between each EMA prompt.

### Schedule

The majority of studies (9 out of 13) monitored participants during one period of time (ie, one wave of data collection), while other studies included up to 8 waves of data collection. The duration of each monitoring period included: 2 days (1 study), 4 days (6 studies), 7 days (5 studies), and 14 days (1 study). Studies with more than one monitoring period had smaller durations than those with only one monitoring period. Typically, the shorter the duration of the study, the higher the prompt frequency per day. For example, one study prompted participants 44 times on weekdays and 68 times on weekends during a 4-day monitoring period [17]. The studies with the longest durations prompted their participants 4 times per day for 14 consecutive days [15].

Seven of the reviewed studies had different prompting frequencies for weekdays and weekend days. In general, participants received more prompts during weekend days than weekdays. Prompting frequency ranged from 2 times per day (during weekdays) to 68 times per day (during weekend days), with the median being 7 times per day. The majority of the studies (9 out of 13) did not collect EMA data during school hours (eg, between 8am-3pm). There were several studies conducted by the same group of researchers that used the same prompting frequency schedule across studies: 44 prompts per day during weekdays and 68 prompts during weekend days [14,15,26,28]. Prompt frequencies varied significantly between studies that employed paper and pencil as compared with electronic data collection tools. For example, a paper-and-pencil study [18] utilized a prompt frequency of 7 times per day, while an electronic data collection EMA study prompted participants 20-30 times per day [16].

### Technology and Administration

A majority of the studies (7 out of 13) used electronic EMA methods and the rest of the studies used paper-and-pencil-based diary methods. For studies that used electronic EMA methods, 4 used cellular phones and 3 used handheld computers. Only one study used a combination of technology for the EMAs. This study divided the sample into two equal groups: one group completed EMAs via paper-based diary and the other group completed EMAs via cellular phones [22]. With the exception of one study [16], studies that used electronic devices for the EMAs had relatively small sample sizes (*n*<175). Training sessions on the use of the EMA technology for participants were held in two studies. One study used parent-reported dietary consumption data for children aged 1.5-10 years old [18]; all other studies collected the self-reported data directly from the youth.

### Prompting Strategy

Most (11 out of 13) of the studies used interval-contingent prompting strategy. Of these 11 studies, 5 used fixed interval contingent only (eg, every 15 minutes), 3 used random interval contingent only (eg, randomly within a 2-hour block), and 3 used combined strategies (eg, event based and interval contingent, interval contingent and evening report). One study used event-based strategy only for collecting EMA responses.

The sampling strategy used by the studies seemed to be related to the behavior of interest. All but one study that measured physical activity used interval contingent sampling, while the majority of studies measuring nutrition habits used events-based sampling in their design.

### Response and Compliance

Table 2 summarizes the response and compliance-related results for all studies. Although most studies reported participant initial enrollment, only 2 studies formally reported attrition rate [19,20]. Another 9 studies reported their respective analytical sample size, although most of the studies did not clearly indicate why the analytical sample size varied from the initial enrollment (eg, participant attrition, device malfunction, or other reasons).

For the studies that utilized an interval-contingent prompting strategy via electronic devices, none of the studies reported how many prompts were actually received by participants. Eight studies did not report the average number of percentage of EMA prompts answered by participants. No study reported reasons for unprompted or unanswered prompts.

Among studies that reported compliance, compliance rates were relatively high (mean=71.3%), with reported compliance ranging from 43.8-95.9%. Compliance reporting differed for paper-and-pencil and electronic EMA designs. Only 2 (out of 6) paper-and-pencil designs reported compliance [26], whereas all of the electronic designs reported compliance rates. Results from Berkman et al compared compliance between paper-and-pencil and electronic EMAs, and reported that the electronic group was more compliant than the paper-and-pencil group (95.9 and 69.9%, respectively) [22]. Even though several studies had more than one monitoring period, no studies reported compliance by wave. One study reported compliance by day [20] and reported that daily average compliance rates declined from 63% at the start of the study to 23% on day 7, demonstrating a decline in answered EMA prompts as the monitoring period progressed.

Only 3 studies reported latency (the time period between when participants receive an EMA prompt and when the EMA is answered) of participant responses. In order to ensure the momentary nature of the responses, 2 electronically administered EMA studies designed their EMAs to prohibit responses 4 minutes [16] or 8 minutes [19] after signaling prompts were sent. No studies reported on why respondents were late responding to prompts.

**Table 2:** Ecological momentary assessment (EMA) response and compliance-related results from nutrition and physical activity studies in youth.

Citation	Initial enrollment^a^	Analytical sample size^b^	Average answered EMA survey prompts (per participant) M (SD)^c^	Average compliance rate^e^	Average latency (>15 minutes)^f^
Berkman et al [22]	44	NR^f^^g^	NR^f^^g^	Electronic: 96% Paper and pencil: 70%	Electronic: 40.1% Paper and pencil: 73.2%
Biddle et al [14]	991	948	NR^f^^g^	NR^f^^g^	71.7%
Biddle et al [15]	623	550	NR^f^^g^	NR^f^^g^	NR^f^^g^
Carels et al [23]	30	NR^f^^g^	Lapses: 11.8 (10.9) Temptations: 8.7 (8.3) Random prompts: 18.3 (8.3)	NR^f^^g^	N/A
Dunton et al [16]	568	524	24.3 (3.4)	83% (SD=9.4)	0%
Dunton et al [24]	121	108	31.2^a^	78%	NR^f^^g^
Gorely et al [17]	1604	1371	NR^f^^g^	50%	74.1%
Grenard et al [25]	158	158	Random: 11.8^a^ Eating events: 13.4^a^ Evening report: 6.58^a^	Random: 71% Evening reports: 95%	NR^f^^g^
Mak et al [18]	-	642	N/A	NR^f^^g^	N/A
Rouse et al [26]	147	84	NR^f^^g^	57%	NR^f^^g^
Rusby et al [19]	82	80	74.9^a^	Total: 69%^a^	0%
Spook et al [20]	30	30	4.3	44%	NR^f^^g^
Thomas et al [27]	43	39	31.3%^a^	71%	NR^f^^g^

^a^Initial enrollment: number of participants who consented to the study.

^b^Analytical sample size: number of participants in the main analysis.

^c^Average answered EMA survey prompts (per participant): average of number of survey prompts each participant responded to.

^d^Average compliance rate: average of number of answered surveys out of total planned EMA surveys per participant, can include compliance for each monitoring period.

^e^Average latency (>15 minutes): the average time between prompting to participants answered the prompt.

^f^Numbers were hand calculated from information available.

^g^NR^g^: not reported in paper.

## Discussion

The primary aim of this study was to systematically review the literature on EMA methods and procedures relating to nutrition and physical activity in youth in order to describe the common practices in EMA methodologies, and to identify response and compliance rates for this target population group. There has been very limited research using EMA methodology to assess youth nutrition and physical activity behaviors. A total of 13 individual EMA studies met inclusion criteria for this review and varied considerably in methodological and results reporting strategy. Enhancements to design and reporting may increase the interpretability and generalizability of EMA findings, application to intervention projects, and ease of use when assessing nutrition and physical activity among youth.

Overall, a significant amount of key information was not reported from studies that were included in this review, demonstrating the need for a reporting guideline that is tailored to the unique features of EMA studies, especially studies that utilize electronic devices. On the basis of results from this review and building on existing guidelines [13], CREMAS was developed to provide recommendations in reporting future EMA studies (Table 3). These recommendations to unify reporting include 16 items that address various sections in a manuscript, and in general, could be applied to EMA studies across disciplines.

**Table 3:** An adapted STROBE Checklist for Reporting EMA Studies (CREMAS).

Topic	Item #	Checklist item	Page number reported
Title								
		1	Include ecological momentary assessment in title and key words	
Introduction								
	Rationale	2	Briefly introduce the concept of EMA and provide reasons for utilizing EMA for this study or topic of interests (eg, to examine time-varying predictors of unhealthy eating occasions in children’s daily lives)	
Methods^a^								
	Training	3	Indicate if, and by what methods, training of participants for EMA protocol was used	
	Technology	4	Describe what technology, if any, was used. Include the following information: device (eg, mobile phone, portable computer), model (eg, Nexus 4, iPod), operating system (eg, Android, Windows), and EMA program name	
	Wave duration	5	State the number of waves for the study (eg, 2 monitoring periods over the course of 1 year)	
	Monitoring period	6	State the number of days each wave of the study lasted, and how many weekdays versus weekend days	
	Prompting design	7	Indicate the prompting strategy used for the study (eg, event-based, interval-based, or a combination of the two). If using interval-based strategy, indicate what type of schedule is used (eg, fixed, random, or hybrid interval)	
	Prompt frequency	8	Intended frequency of prompts per day. Break down by weekdays and weekend days if applicable	
	Design features	9	Describe any design feature to address potential sources of bias (eg, reactivity) or participant burden (eg, EMA questions appearing in different orders)	
Results^a^								
	Attrition	10	Indicate participant attrition throughout the study; report attrition rates both by monitoring days and waves, if applicable	
	Prompt delivery	11	Report number of EMA prompts that were planned to be delivered. If possible, also report the number of EMA prompts that were actually received by participants and indicate reasons for why prompts were not sent out (eg, technical issues or participant noncompliance reason such as phone was powered off)	
	Latency	12	Report the amount of time from prompt signal to answering of prompt	
	Compliance rate	13	Report total answered EMA prompts across all subjects and the average number of EMA prompts answered per person. Report compliance rate both by monitoring days and waves, if applicable. Indicate reasons for noncompliance, if known	
	Missing data	14	Report whether EMA compliance is related to demographic or time-varying variables	
Discussion								
	Limitations	15	Discuss limitations of the study, taking into account sources of potential bias when using EMA methods (eg, reactivity, use of technology)	
	Conclusions	16	Provide a general interpretation of results and discuss the benefits of using EMA (eg, improving understanding of daily behaviors)	

^a^Secondary data analysis paper can refer to a main methods paper that has discussed all of these items.

This review shows that studies have used both paper-and-pencil and electronic EMA designs to capture nutrition and physical activity in youth. Compared with paper-and-pencil design, there are several benefits to using electronic EMA designs such as automatic prompt signaling (eg, auditory or tactile), instant data transfer via download or Internet-based secure servers, and greater accessibility and convenience for participants [29]. More importantly, electronic EMA collection instruments are able to collect exact times of each assessment and ensure that the assessments are completed following study protocols. Indeed, several studies have shown that even with signaling prompts and detailed instructions, the completion of paper-and-pencil EMAs may not occur in real time [30].

Although technologies can assist in making the delivery of EMA surveys more systematic, they also have some limitations. For example, EMA surveys may fail to be delivered because of technological issues (eg, problems with the app) or user compliance issues (eg, subjects can have phone turned off). Therefore, it is important that authors report the intended number of EMA prompts and the actual number of EMA prompts participants received if possible. The utilization of electronic EMA devices may pose some challenges for some studies. For example, the electronic devices themselves can be a costly research expense. The majority of the reviewed studies that used electronic devices provided those devices to the participants (instead of participants using their own device), which ensured the consistency of the usability and functionality in the administration of EMAs. However, given the cost associated with providing loaned devices, drawbacks, such as limiting sample size and participant burden of remembering to keep the study device with them and charged, should be considered. Often, an experienced computer programmer and several rounds of pilot testing are needed to develop electronic EMAs to be administered on mobile phones or personal digital assistants (PDAs). Nevertheless, free open source EMA programs (eg, PACO by Google, MovisensXS by Movisens GmbH) are available and can be tailored to researchers’ specifications.

In general, response and compliance-related data were inconsistently reported. This information is critical to assess the quality of data collected by a study. More importantly, these data will provide valuable information for future studies planning to adopt EMA methods in optimizing study design (eg, Will compliance rate be very different between a study that delivers 4 prompts a day versus a study that delivers 44 prompts a day?). This review is not able to answer this question fully because over half of the reviewed studies did not report compliance data. Therefore, it is highly recommended that all future EMA studies report response and compliance-related data, except for studies that only utilize event-based design with manually initiated reporting since there is no set number of diary entries or prompts that participants are required to complete. In addition, the majority of studies did not report latency. Due to the in-the-moment nature of EMA studies, it is critical that EMAs are completed shortly after prompts are received. One way to ensure the momentary nature of the responses is limit the time respondents have to complete the EMA, as was done by two studies included in this review [16,19].

Although there is no consistently agreed upon gold standard for acceptable rates of compliance to EMAs, Stone and Shiffman noted that if compliance falls below 80% there may be concern that data are not representative or generalizable to participants’ usual daily lives [12]; however, reasons for missing data (random vs. not random) should be taken into consideration. Thus, we encourage future EMA studies to report reasons for noncompliance or missing data whenever possible. Regardless of compliance rate, missing EMA data (prompted and unprompted) should be examined for systematic associations with known temporal (eg, time of day, day of the week, chronological day in study, study wave) and demographic (eg, age, gender, race/ethnicity, SES, adiposity) factors [31,32]. A more thorough analysis of missing EMA data would include examining whether the rates and likelihood of unanswered EMA prompts are associated with information provided by temporally adjacent available EMA data (eg, average daily levels, levels reported at EMA prompts before or after the unanswered prompt). Pattern-mixture random-effects regression modeling offers a promising strategy for understanding missingness patterns with EMA data [33]. For data determined to be missing at random (MAR) or missing completely at random (MCAR) (ie, associated with unobserved or observed variables), imputation methods should be considered [34]. With consistent reporting of response and compliance rates, audiences would be able to determine whether the data may be generalizable to all days of the week, times of day, or situations throughout the day.

Even though EMAs offer many methodological benefits, there are still some challenges when utilizing real-time data capture methods. Although most EMA studies aim to observe participants’ behavior without influencing it, repetitive exposure to EMA items relating to nutrition and physical activity may trigger participants to adjust behaviors in ways they otherwise would not. Evidence suggests that the mere act of measuring a behavior could have some impact on that behavior in the future [35]. Further, if EMA prompting rates are too frequent and/or EMA questions are too repetitive, participants may opt not to respond to the surveys or drop out of the study altogether. The study with the highest frequency of prompting (44 prompts during weekdays and 68 prompts during weekend days) also reported the lowest compliance rate at 57% [26]. To reduce concerns about participant reactivity and burden, researchers should aim to use the fewest number of prompted surveys possible to answer their questions or interests.

Researchers could also consider combining EMAs with other objective measurement to capture the behaviors of interests. For example, Dunton and colleagues used electronic EMA in combination with accelerometry to measure children’s physical activity [21]. In this case, the accelerometry device can continuously measure activity intensity while EMAs can be used to capture other information such as type of activity, and contextual information of activities (eg, where and with whom).

Overall, the lack of consistency in reporting EMA methods greatly limits the scientific impact and possible use of findings for behavioral assessments or development of intervention strategies for nutrition and physical activity behaviors in youth. A clear and detailed report of EMA design features could be very helpful for researchers that are new to EMA methodologies. Consistently reporting these types of data will also be useful for future researchers to understand which device/model/systems are effective for nutrition and physical activity assessment studies. Without providing important aspects of EMA design and results, data can be misinterpreted. Researchers may also want to report intrapersonal (person-level) compliance rates, as there might be significant individual variation. In general, reporting more complete aspects of EMA data will help the audience to fully interpret the results, including generalizability and application to future EMA designs.

### Limitations

Although this review is unique in that it is the first to examine EMA studies of nutrition and physical activity behaviors among youth, it has several limitations. First, we attempted to be exhaustive in the literature search, but it is possible that some studies may have been missed. Second, since reporting strategies were so diverse, our ability to report quantitative information was limited. Further, for total EMA prompts received and answered, latency, compliance, and attrition rates, so much data was missing across studies that it was hard to make intuitive interpretations of these results.

### Conclusions

This review presented the data of key EMA methods from 13 nutrition and physical activity studies. Utilizing EMA methods to study nutrition and physical activity in young people has many powerful benefits, including ecological validity and minimizing retrospective response bias. However, based on our review, many studies fail to employ all the features of EMA methods, as described by Shiffman and colleagues [5], and reporting strategies are inconsistent and insufficient. In order to maximize the impact that EMA data has in the scientific literature, reporting needs to be systematic across studies, allowing greater interpretability and reach of EMA methodologies. Therefore, in order to adequately interpret findings from EMA studies, several items need to be included when reporting EMA methods and results; we created a checklist for others to use. Reporting these key methodological EMA data can enhance efficacy, reliability, and validity of study findings and may lead to increased understanding and interpretation of results.

## Abbreviations

CREMAS: Checklist for Reporting EMA Studies
EMA: ecological momentary assessment
MAR: missing at random
MCAR: missing completely at random
PA: physical activity
PDA: personal digital assistant
PRISMA: Preferred Reporting Items for Systematic Reviews and Meta-Analyses
STROBE: Strengthening the Reporting of Observational Studies in Epidemiology

## References

[1] Ogden CL, Carroll MD, Curtin LR, Lamb MM, Flegal KM (2010). Prevalence of high body mass index in US children and adolescents, 2007-2008. JAMA.

[2] Kann L, Kinchen S, Shanklin SL, Flint KH, Hawkins J, Harris W, Lowry R, O'Malley Olsen E, McManus T, Chyen D, Whittle L, Taylor E, Demissie Z, Brener N, Thornton J, Moore J, Zaza S (2013). Center for Surveillance, Epidemiology, Laboratory Services, CDC.

[3] Bridger T (2009). Childhood obesity and cardiovascular disease. Paediatr Child Health.

[4] Sweat V, Bruzzese J, Albert S, Pinero DJ, Fierman A, Convit A (2012). The Banishing Obesity and Diabetes in Youth (BODY) Project: description and feasibility of a program to halt obesity-associated disease among urban high school students. J Community Health.

[5] Shiffman S, Stone AA, Hufford MR (2008). Ecological momentary assessment. Annu Rev Clin Psychol.

[6] Stone AA, Broderick J (2007). Real-time data collection for pain: appraisal and current status. Pain Med.

[7] Schwarz N (2007). Retrospective and concurrent self-reports: the rationale for real-time data capture. The science of real-time data capture: self-reports in health research.

[8] Dunton GF, Segel CM (2011). Using real-time data capture methods to investigate children's physical activity and eating behaviors. Childhood obesity: risk factors, health effects and prevention.

[9] Liao Y, Intille SS, Dunton GF (2015). Using ecological momentary assessment to understand where and with whom adults' physical and sedentary activity occur. Int J Behav Med.

[10] Liao Y, Shonkoff ET, Dunton GF (2015). The Acute Relationships Between Affect, Physical Feeling States, and Physical Activity in Daily Life: A Review of Current Evidence. Front Psychol.

[11] Pickering TA, Huh J, Intille S, Liao Y, Pentz MA, Dunton GF (2016). Physical activity and variation in momentary behavioral cognitions: an ecological momentary assessment study. J Phys Act Health.

[12] Stone AA, Shiffman S (2002). Capturing momentary, self-report data: a proposal for reporting guidelines. Ann Behav Med.

[13] von Elm E, Altman DG, Egger M, Pocock SJ, Gøtzsche PC, Vandenbroucke JP (2007). The Strengthening the Reporting of Observational Studies in Epidemiology (STROBE) statement: guidelines for reporting observational studies. Prev Med.

[14] Biddle SJ, Gorely T, Marshall SJ, Cameron N (2009). The prevalence of sedentary behavior and physical activity in leisure time: a study of Scottish adolescents using ecological momentary assessment. Prev Med.

[15] Biddle SJ, Soos I, Hamar P, Sandor I, Simonek J, Karsai I (2009). Physical activity and sedentary behaviours in youth: data from three Central-Eastern European countries. Eur J Sport Sci.

[16] Dunton GF, Whalen CK, Jamner LD, Floro JN (2007). Mapping the social and physical contexts of physical activity across adolescence using ecological momentary assessment. Ann Behav Med.

[17] Gorely T, Marshall SJ, Biddle SJ, Cameron N (2007). The prevalence of leisure time sedentary behaviour and physical activity in adolescent girls: an ecological momentary assessment approach. Int J Pediatr Obes.

[18] Mak TN, Prynne CJ, Cole D, Fitt E, Roberts C, Bates B, Stephen AM (2013). Assessing eating context and fruit and vegetable consumption in children: new methods using food diaries in the UK National Diet and Nutrition Survey Rolling Programme. Int J Behav Nutr Phys Act.

[19] Rusby JC, Westling E, Crowley R, Light JM (2014). Psychosocial correlates of physical and sedentary activities of early adolescent youth. Health Educ Behav.

[20] Spook JE, Paulussen T, Kok G, Van Empelen P (2013). Monitoring dietary intake and physical activity electronically: feasibility, usability, and ecological validity of a mobile-based ecological momentary assessment tool. J Med Internet Res.

[21] Dunton GF, Liao Y, Intille SS, Spruijt-Metz D, Pentz M (2011). Investigating children's physical activity and sedentary behavior using ecological momentary assessment with mobile phones. Pediatr Obes.

[22] Berkman ET, Giuliani NR, Pruitt AK (2014). Comparison of text messaging and paper-and-pencil for ecological momentary assessment of food craving and intake. Appetite.

[23] Carels RA, Hoffman J, Collins A, Raber AC, Cacciapaglia H, O'Brien WH (2001). Ecological momentary assessment of temptation and lapse in dieting. Eat Behav.

[24] Dunton GF, Intille SS, Wolch J, Pentz MA (2012). Children's perceptions of physical activity environments captured through ecological momentary assessment: a validation study. Prev Med.

[25] Grenard JL, Stacy AW, Shiffman S, Baraldi AN, MacKinnon DP, Lockhart G, Kisbu-Sakarya Y, Boyle S, Beleva Y, Koprowski C, Ames SL, Reynolds KD (2013). Sweetened drink and snacking cues in adolescents: a study using ecological momentary assessment. Appetite.

[26] Rouse PC, Biddle S (2010). An ecological momentary assessment of the physical activity and sedentary behaviour patterns of university students. Health Education Journal.

[27] Thomas JG, Doshi S, Crosby RD, Lowe MR (2011). Ecological momentary assessment of obesogenic eating behavior: combining person-specific and environmental predictors. Obesity (Silver Spring).

[28] Gorely T, Marshall SJ, Biddle SJ, Cameron N (2007). Patterns of sedentary behaviour and physical activity among adolescents in the United Kingdom: Project STIL. J Behav Med.

[29] Heron KE, Smyth JM (2013). Body Image Discrepancy and Negative Affect in Women's Everyday Lives: An Ecological Momentary Assessment Evaluation of Self-Discrepancy Theory. Journal of Social and Clinical Psychology.

[30] Broderick JE, Schwartz JE, Shiffman S, Hufford MR, Stone AA (2003). Signaling does not adequately improve diary compliance. Ann Behav Med.

[31] Little R, Rubin D (2002). When questions change behavior: the role of ease of representation. Statistical analysis with missing data.

[32] Schafer J (1997). Analysis of incomplete multivariate data.

[33] Hedeker D, Rose JS (2000). The natural history of smoking: a pattern-mixture random-effects regression model. Multivariate applications in substance use research: new methods for new questions.

[34] Sinharay S, Stern HS, Russell D (2001). The use of multiple imputation for the analysis of missing data. Psychol Methods.

[35] Levav J, Fitzsimons GJ (2006). When questions change behavior: the role of ease of representation. Psychol Sci.

